# circ_000166/miR-296 Aggravates the Process of Diabetic Renal Fibrosis by Regulating the SGLT2 Signaling Pathway in Renal Tubular Epithelial Cells

**DOI:** 10.1155/2022/6103086

**Published:** 2022-05-16

**Authors:** Sheng Chen

**Affiliations:** Department of Nephrology, Ningbo Medical Center Lihuili Hospital, Ningbo, Zhejiang 315000, China

## Abstract

Diabetic renal fibrosis is a common cause of end-stage renal disease, and the circRNA-miRNA-mRNA network may play an important role in the progression of diabetic nephropathy- (DN-) induced renal fibrosis. In this study, the role of circ_000166/miR-296/SGLT2 in the process of DN-related renal fibrosis was studied by constructing an animal model of DN renal fibrosis via lentiviral transfection, plasmid transfection, and dual-luciferase reporting techniques. Compared with that of normal controls, the expression of circ_000166 in the kidney tissues of DN renal fibrosis mice substantially increased. Silencing circ_000166 could minimize kidney damage and decrease urine protein levels, thereby inhibiting the progression of renal fibrosis. Moreover, circ_000166 could act as the ceRNA of miR-296 and competitively bind to miR-296, leading to an increase in the expression of the SGLT2 gene regulated by miR-296. Through mutual verification via in vivo and in vitro experiments, miR-296 was overexpressed and SGLT2 was silenced. Results showed that DN renal fibrosis and cell apoptosis were considerably reduced. We postulate that circ_000166/miR-296/SGLT2 may become a new target in the progression of DN renal fibrosis, and the regulation of this pathway may be a promising strategy for clinical treatment of DN renal fibrosis.

## 1. Introduction

Diabetic nephropathy (DN) is one of the most common microvascular complications among patients with diabetes and an important cause of disability and death [1, 2]. It is characterized by excessive accumulation of the extracellular matrix (ECM), thickening of the glomerulus and tubular basement membrane, and increase in the mesangial matrix, features that eventually develop into glomerular sclerosis and tubular interstitial fibrosis [3, 4].

Noncoding RNA (ncRNA) includes microRNA (miRNA), long ncRNA (lncRNA), and circular RNA (circRNA). Numerous studies reported that ncRNA plays a key role in various human diseases [5–7]. The present study investigated the interaction between miRNA and circRNA. miRNA can induce gene silencing by binding to the 3′-untranslated region of target mRNA, thereby inducing translational inhibition. The biological effects of circRNA are mainly mediated by miRNA [8, 9]. circRNA is a new type of ncRNA characterized by a covalent closed loop [10]. circRNA can be used as one of the competing endogenous RNAs (ceRNA) that can competitively bind to miRNA, thereby blocking the translation or inducing the degradation of the target mRNA [11]. When miRNA is competitively bound by circRNA, the level of mRNA transcription regulated by miRNA will increase [1]. A recent study demonstrated that circRNA_000166 can promote the proliferation, migration, and invasion of colon cancer cells by upregulating the expression of ELK1 in miK-330-5p sponge [12].

Diabetic renal fibrosis is caused by the unbalanced metabolism of ECM molecules that may eventually lead to renal failure. Previous studies reported that circ-AKT3 inhibits ECM accumulation in mesangial cells of DN by regulating miR-296-3p/E-cadherin signals [13]. miRNA imbalance is closely related to the occurrence of diabetic renal fibrosis. Moreover, circRNA can act as a real sponge of miRNA to regulate the expression of the target gene. Thus, the circRNA-miRNA-mRNA network may play a very important role in the process of renal fibrosis caused by DN [14].

Sodium-glucose cotransporter 2 (SGLT2) is a protein mainly expressed in the apical membrane of the proximal tubule of the kidneys, and 90% of the glucose filtered by the glomerulus can be reabsorbed by this protein [15, 16]. Increased glucose uptake by cultured proximal renal tubular cells through SGLT2 can cause proapoptotic effects [17]. Apoptosis of renal tubular epithelial cells and atrophy of renal tubules eventually lead to tubular interstitial fibrosis [18].

This study investigated the role of the circ_000166/miR-296/SGLT2 network in the process of diabetic renal fibrosis. Elucidating the circ_000166/miR-296/SGLT2 regulatory network will help us to better understand the molecular mechanisms of diabetic renal fibrosis and recognize how they may serve as new biomarkers of DN and potential therapeutic targets.

## 2. Materials and Methods

### 2.1. Animals and Exposures

All animal experiments of this study were approved by the experimental animal ethics committee of Affiliated Hospital of Weifang Medical University. Male C57BL/6J mice (Beijing HFK Biotechnology Co., Ltd.) (8 weeks old, 25 ± 1 g in weight) were raised in a special-grade sterile animal room at 23 ± 1°C in a 12-hour day-night cycle. All mice had free access to food and water. Fourteen C57BL/6J mice were selected as normal controls and fed with a standard diet (8% fat). The other groups of mice were fed with a high-fat diet feed (40% fat) for 8 weeks. Afterward, the mice in the DN model group were intraperitoneally injected with 50 mg/kg streptozotocin (STZ, Sigma Chemicals, St. Louis, MO) dissolved in 100 mM citrate buffer (pH 4.5) for five consecutive days. The mice were then anesthetized with pentobarbital sodium and then killed using a carbon dioxide treatment device. The mice in the control group received an equal volume of citrate buffer. The mice with fasting blood glucose > 12 mmol/L were designated as the T1DM model mice (blood was drawn from the tail vein to measure plasma glucose via standard laboratory methods). The DN standard was 24 h UMA level ≥ 30 mg. Renal fibrosis was assessed via renal histopathology.

### 2.2. Cell Cultures and Exposure

Renal tubular epithelial cells (HK-2, American Type Culture Collection, Rockville, Maryland, USA) were cultured in DMEM/F12 medium containing 10% FBS (Gibco) at 37°C. The HK-2 cells were inoculated in a 12-well plate and cultured in a constant-temperature incubator at 37°C for 16–24 h. When the cell confluence reached 30%, the medium was replaced, and HiTransG P infection solution and the corresponding amount of virus (20 *μ*L/well) were added. After 16 h, the medium was changed into a regular medium and the culture was continued. The groups consisted of the mimic-NC group, miR-296 mimic group, inhibitor-NC group, and miR-296 inhibitor group. The expression of miR-296 and SGLT2 in each group was detected 72 h after infection. HK-2 cells were seeded into a 12-well plate. When the cells reached 70% confluence, si-NC, si-circ_000166, vector-NC, and vector-circ_00016 (GenePharma Co Ltd., Shanghai, China) were transfected into the HK-2 cells. Lipofectamine 2000 (11668027, American Thermoelectric) and siRNA were premixed in Opti-MEM (31985062, American Thermoelectric) and then transfected into the cells following the manufacturer's instructions. At 24 h after transfection, the expression of miR-296 and SGLT2 in each group of cells was detected.

### 2.3. In Vitro Cell Model

When the cells were fused to 80%, the medium was replaced with serum-free DMEM low-glucose medium. The serum-free medium was discarded after 24 h of synchronization. The cells were divided into a normal group (D-glucose concentration in medium: 5.5 mmol/L) and a high-glucose group (D-glucose concentration in high-glucose medium: 25 mmol/L). Each group was provided with three holes. The cells were cultured for 48 h in an incubator at 37°C. Afterward, the cells were collected for subsequent tests.

### 2.4. Lentiviral Transfection

Lentivirus carrying the si-NC and si-circ_000166 target genes, as well as sh-NC and sh-SGLT2 lentivirus expression vectors (synthesized by Jiangsu Genechem Technology Co., Ltd.), was constructed. The animals were transfected by disinfecting the abdomen and then anesthetizing the mice in each group via intraperitoneal injection of 40 mg/kg sodium pentobarbital. Subsequently, the abdominal cavity was opened from the midline of the abdomen to expose both kidneys. A 29-gauge syringe was used to slowly inject 150–200 *μ*L of a solution of the lentiviral vector (titer 1 × 10^6^ CFU/mL) carrying the corresponding target gene into the kidney parenchyma. The incision was closed, and the mice were returned to the cage to breed. The mice were sacrificed on the 28th day, and their kidneys were removed for later use. The lentivirus was transfected by adding 2 mL of fresh medium containing 6 *μ*g/mL polybrene to HK-2 cells and adding an appropriate amount of virus suspension. The cells were incubated at 37°C. After 24 h, `the virus-containing medium was replaced with fresh medium. After 72 h of continuous cultivation, the expression of fibrosis-related factors in each group was detected.

### 2.5. Dual-Luciferase Assay

The luciferase reporter gene test was performed using the dual-luciferase reporter assay system (Promega, USA) following the manufacturer's protocols. HK-2 cells (1.5 × 10^4^/well) were inoculated into a 96-well plate. After 12 h, the cells were transiently cotransfected with the pRL-TK plasmid (Promega) containing the Renilla luciferase gene for internal standardization and various constructs containing pMIR-circ_000166 and pMIR-circ_000166-mut. At 36 h after transfection, the cells were lysed, and luciferase activity was measured. Finally, 100 *μ*L of the protein extract was analyzed using a luminometer. All experiments were conducted at least three times.

### 2.6. qRT-PCR

HK-2 cells were lysed with the TRIzol reagent (9108, TaKaRa, Biotech, Japan), and total RNA was extracted therefrom in accordance with the manufacturer's instructions. The total RNA was reverse-transcribed (AT301-03, TransGen Biotech, Beijing, China), and the samples were normalized. The expression levels of circ_000166, collagen IV, TGF-*β*1, ACE, AT1, VEGF, miR-296, SGLT2, GAPDH, and U6 were detected using the TransStart Top Green qPCR SuperMix kit (AQ131-04, TransGen Biotech, Beijing, China). circ_000166, hsa_circ_000166, and miR-296 were standardized with U6, whereas the others were standardized with GAPDH. Expression analysis was calculated via the comparative Ct method. The primers used were as follows: GAPDH: forward: CCACATCGCTCAGACACCAT, reverse: CCAGGCGCCCAATACG; U6: forward: CGCTTCGGCAGCACATATAC, reverse: TTCACGAATTTGCGTGTCAT; circRNA_000166: forward: CCATATTGAATCACAGTGCGT, reverse: ACAGCGCAGTAAGGTGCTCG; miR-296: forward: TGCCTAATTCAGAGGGTTGG, reverse: CTCCACTCCTGGCACACAG; TGF-*β*1: forward: GAGGCGGTGCTCGCTTTGTA, reverse: CGTTGTTGCGGTCCACCATTA; ACE: forward: ACGAGCACGACATCAACTTCCTCA, reverse: AGTAGTTCATCATGGCCGAGGCT; AT1: forward: AGGATGACTGTCCCAAAGCTGGAA, reverse: ACGTTTCGGTGGATGATAGCTGGT; VEGF: forward: AGCACAGCAGATGTGAATGC, reverse: AATGCTTTCTCCGCTCTGAA; collagen IV: forward: AATCCCAGGAGGACGAGGTGT, reverse: GGATTACCCACTTGCCCCCAG; and SGLT2: forward: CTCCGGAGCTGTATTCATCCA, reverse: AGCCCTCCTGTCACCGTGTA.

### 2.7. Flow Cytometry

The cells were collected and then centrifuged. Each group of cells were then turned into pellets and divided into groups. Afterward, 500 *μ*L of binding buffer was added to suspend the cells, and then, 5 *μ*L of Annexin V-EGFP was added. The solution was then mixed well. Subsequently, 5 *μ*L of propidium iodide was added and mixed well. The mixture was allowed to react at room temperature in the dark for 5–15 min. Cell apoptosis was detected via flow cytometry at excitation wavelength of 488 nm and emission wavelength of 530 nm.

### 2.8. Western Blotting

A cell lysate (Beyotime, China) containing RIPA and PMSF (100 : 1) was used to extract tissue proteins. The tissue protein extract was analyzed via sodium dodecyl sulfate polyacrylamide gel electrophoresis (SDS-PAGE), transferred onto a PVDF membrane, and then analyzed via western blotting. The antibodies incubated for western blotting were collagen IV (ab6586, Abcam), TGF-*β*1 (ab215715, Abcam), ACE (SC-2908, Santa Cruz, CA), AT1 (SC-2225, Santa Cruz, CA), VEGF (SC-152, Santa Cruz, CA), and GAPDH (AB-P-R001, Abcam). The extract was incubated with the horseradish peroxidase-conjugated anti-rabbit secondary antibody (1 : 5000, ZB-2301, ZSGB-Bio, Beijing, China), and then, its protein bands were determined using an ECL blot detection reagent (WBKLS0500, Millipore Corporation).

### 2.9. Statistical Analysis

Statistical analysis was performed using SPSS 19.0 software. All values are expressed as mean ± standard deviation (SD) of three biological replicates or samples. Data were analyzed via one-way ANOVA or LSD test. *p* < 0.05 was considered statistically significant.

## 3. Results

### 3.1. circ_000166 Is Upregulated in Kidney Tissues of DN Mice

**Figure 1:**
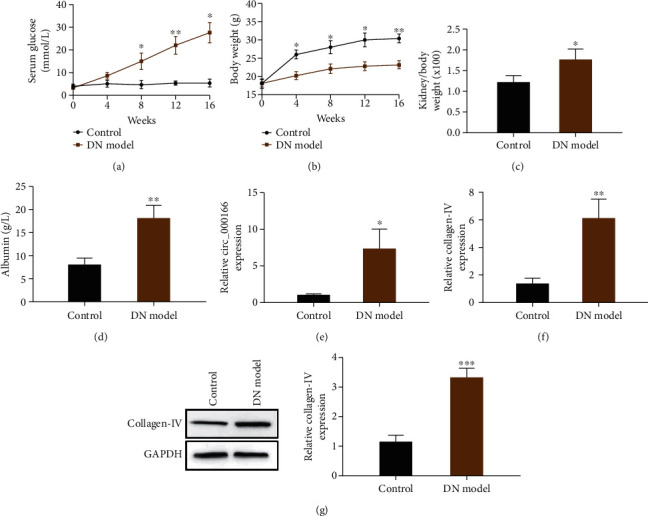
The expression of circ_000166 in the kidney tissue of diabetic renal fibrosis model mice is increased. At 0, 4, 8, 12, and 16 weeks, (a) blood sugar level, (b) weight, (c) the ratio of kidney-to-body weight, and (d) urine protein levels of the normal control group and T1DM group mice were measured. (e) The expression levels of circ_000166 in the kidney tissues of the two groups of mice were detected by qRT-PCR, and (f, g) the expression levels of collagen IV in the kidney tissues of the two groups of mice were detected by qRT-PCR and western blot. The data are expressed as mean ± standard (control group (*n* = 7), DN group (*n* = 7)). ^∗^*p* < 0.05, ^∗∗^*p* < 0.01, and ^∗∗∗^*p* < 0.001.

We performed intraperitoneal injection of STZ to establish a T1DM mouse model. At 0, 4, 8, 12, and 16 weeks, blood glucose levels and weight changes in each group were recorded. The ratio of the kidney-to-body weight and urine protein levels of mice were compared. Compared with those of the NC mice, the blood glucose level (Figure 1(a)), the ratio of the kidney-to-body weight (Figure 1(c)), and the urine protein level (Figure 1(d)) of the T1DM mice significantly increased, and their weight significantly decreased (Figure 1(b)). We further supplemented the renal pathology results of the DN model. The epithelial and stromal structures of renal tubules were disordered in mice after DN modeling compared with those of the control group (Figure S1). We performed qRT-PCR to detect the expression levels of circ_000166 (Figure 1(e)) and collagen IV (Figure 1(f)) in the two groups of mice. Compared with that of the normal control group, the expression of circ_000166 and collagen IV of the T1DM mouse group increased. We conducted western blot to verify the expression of collagen IV in the kidney tissues of the two groups of mice (Figure 1(g)). The results were consistent with those detected by qRT-PCR, indicating that the diabetic renal fibrosis mouse model was successfully modeled.

### 3.2. Knockdown circ_000166 by Lentivirus Can Inhibit the Occurrence and Development of DN

**Figure 2:**
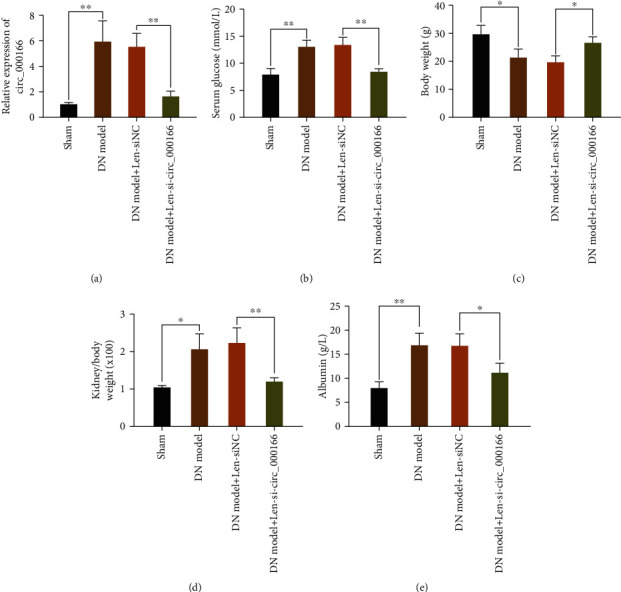
The expression of circ_000166 decreased in the kidney of DN mice. (a) Detect the expression level of circ_000166 in the kidney tissue of each group of mice by qRT-PCR. Compare (b) the blood glucose level, (c) weight, (d) kidney-to-body weight ratio, and (e) urine protein levels of mice between the two groups. All values are expressed as mean ± standard. ^∗^*p* < 0.05, ^∗∗^*p* < 0.01, and ^∗∗∗^*p* < 0.001.

We transfected si-hsa_circ_000166 by injection of lentivirus in the DN model mice. We performed qRT-PCR to detect the expression level of hsa_circ_000166 (Figure 2(a)). We then detected blood glucose levels (Figure 2(b)), body weight changes (Figure 2(c)), kidney-to-body weight ratios (Figure 2(d)), and urine protein levels (Figure 2(e)) of the NC, DN, DN+Len-siNC, and DN+Len-si-hsa_circ_000166 groups, respectively. Compared with those in the NC group, the blood glucose levels, kidney-to-body weight ratios, and urine protein levels in the DN and DN+Len-siNC groups increased. In the DN+Len-si-hsa_circ_000166 group, the blood glucose levels, kidney-to-body weight ratios, and urine proteins were significantly lower than those in the DN and DN+Len-siNC groups, but they increased compared with those in the NC group. However, the opposite trend was observed in weight changes. These results demonstrated that silencing circ_000166 can inhibit DN occurrence and development.

### 3.3. Lentivirus Knockdown circ_000166 Can Inhibit the Process of Diabetic Renal Fibrosis

**Figure 3:**
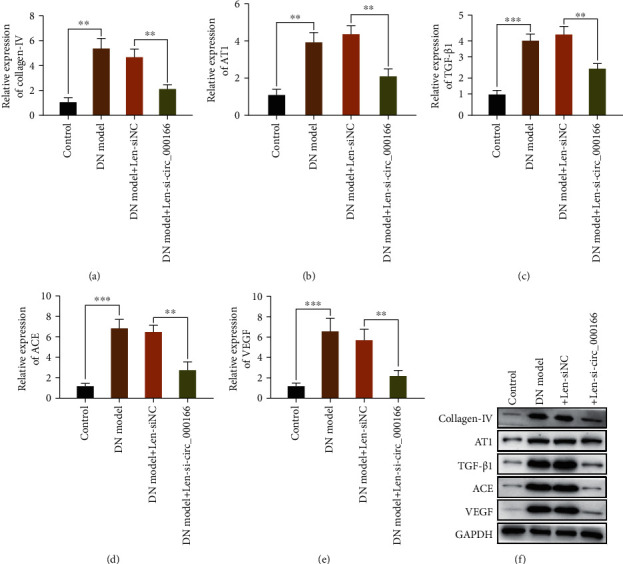
Si-hsa_circ_000166 can inhibit the progression of diabetic renal fibrosis. We used qRT-PCR to detect the expression levels of (a) collagen IV, (b) TGF-*β*1, (c) ACE, (d) AT1, and (e) VEGF in the kidney tissues of mice in each group. (f) The expression levels of the above factors were quantitatively analyzed by western blot. The data are average ± standard (*n* = 10 per group). ^∗^*p* < 0.05, ^∗∗^*p* < 0.01, and ^∗∗∗^*p* < 0.001.

We detected the expression levels of renal fibrosis-related factors, namely, collagen IV, TGF-*β*1, ACE, AT1, and VEGF, via qRT-PCR and western blot (Figures 3(a)–3(f)). Compared with those in the NC group, the expression levels of these factors in the kidneys of mice in the DN and DN+Len-siNC groups increased. Moreover, the expression levels of these factors in the kidneys of mice in the DN+Len-si-hsa_circ_000166 group were significantly lower than those in the DN and DN+Len-siNC groups. These results showed that silencing circ_000166 can inhibit the occurrence and development of diabetic renal fibrosis.

### 3.4. circ_000166 Can Act as a ceRNA for miR-296

**Figure 4:**
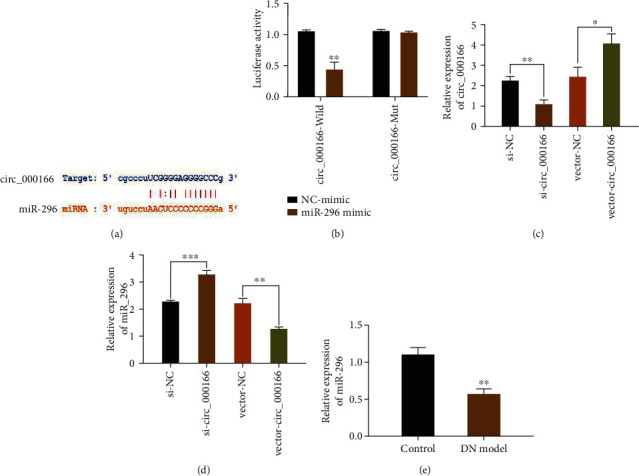
circ_000166 can serve as the ceRNA of miR-296. (a) Sequence of circ_000166 and miR-296 in mouse. (b) The dual-luciferase report detects luciferase activity in mouse kidneys. (c–e) We used qRT-PCR to detect the expression of circ_000166 and miR-296 in each group of mouse kidneys. The data are average ± standard. ^∗^*p* < 0.05, ^∗∗^*p* < 0.01, and ^∗∗∗^*p* < 0.001.

circ_000166 is the target of miR-296, but the circ_000166 mutants are not the target of miR-296 (Figure 4(a)). By constructing a luciferase reporter vector, luciferase and circ_000166 genes were connected and then transfected into cells for detection. Results showed that the miR-296 mimic significantly reduced the relative luciferase activity of circ_000166-Wild but had no significant effect on circ_000166-Mut (Figure 4(b)). The expression level of circ_000166 in different cells was measured via qRT-PCR. After si-circ_000166 treatments, the expression level of circ_000166 significantly decreased, whereas that of miR-296 significantly increased. After treatment with vector-circ_000166, the expression of circ_000166 significantly increased (Figure 4(c)), whereas that of miR-296 significantly decreased (Figure 4(d)). The expression level of miR-296 in the T1DM group was significantly lower than that in the normal control group (Figure 4(e)). These results fully demonstrated that circ_000166 can serve as the ceRNA of miR-296.

### 3.5. SGLT2 as a Target Gene Regulated by miR-296

**Figure 5:**
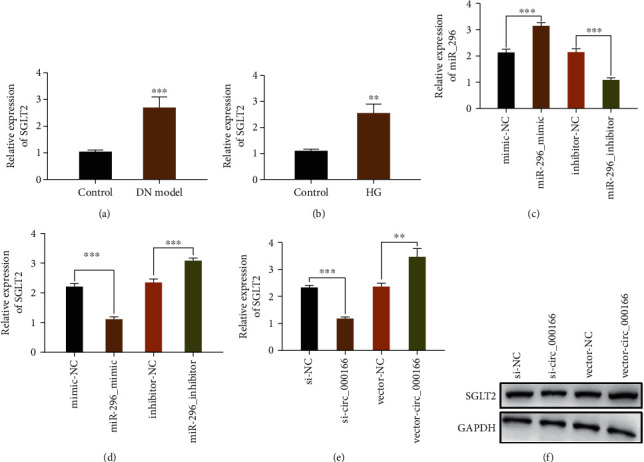
SGLT2 as a target gene regulated by miR-296. (a, b) We used qRT-PCR to detect the expression of SGLT2 in the kidney tissue and the cells of each group. High-glucose group (high-glucose medium containing D-glucose: 25 mmol/L). (c, d QRT-PCR was used to detect the effect of the miR-296 mimic and miR-296 inhibitor on the expression of SGLT2. (e, f) QRT-PCR and western blotting were used to detect the effect of inhibiting circ_000166 on the expression of SGLT2. The data are expressed as mean ± standard. ^∗^*p* < 0.05, ^∗∗^*p* < 0.01, and ^∗∗∗^*p* < 0.001.

Compared with that in the normal control group, the mRNA expression of SGLT2 in the DN and HG groups increased (Figures 5(a) and 5(b)). The miR-296 mimic could increase miR-296 expression and reduce SGLT2 expression, whereas the miR-296 inhibitor could inhibit miR-296 expression and promote SGLT2 expression (Figures 5(c) and 5(d)). Inhibition of circ_000166 could decrease the expression of SGLT2, and vector_circ_000166 could increase the expression of SGLT2 (Figures 5(e) and 5(f)). These results indicated that SGLT2 may be a target gene regulated by miR-296.

### 3.6. Overexpression of miR-296 and Knockdown of SGLT2 Can Reverse the Promotion of Diabetic Renal Fibrosis Regulated by hsa_circ_000166

**Figure 6:**
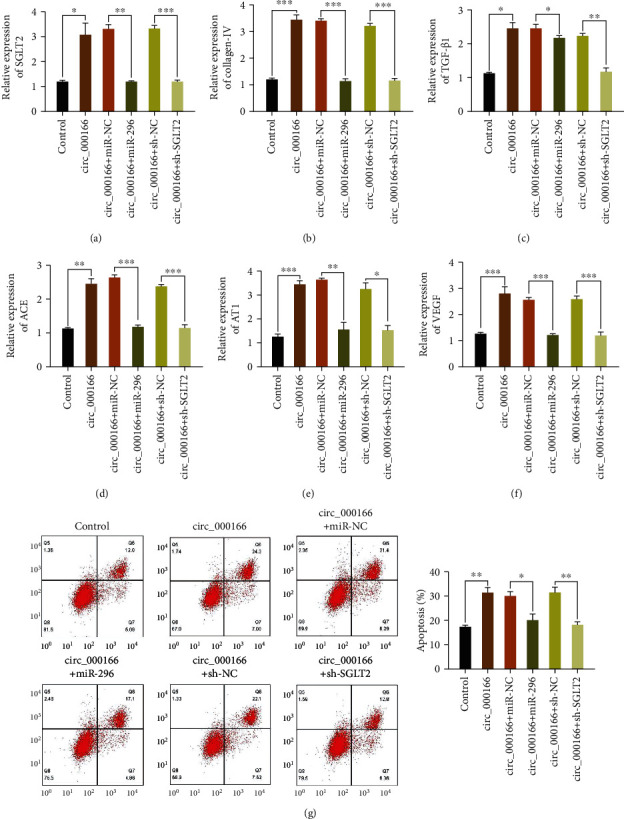
Overexpression of miR-296 and knockdown of SGLT2 reverse the promotion of hsa_circ_000166 on diabetic renal fibrosis. We used qRT-PCR to detect the expression levels of (a) SGLT2, (b) collagen IV, (c) TGF-*β*1, (d) ACE, (e) AT1, and (f) VEGF in each group of cells. Flow cytometry was used to detect apoptosis in each group (g).

circ_000166 was added to the cultured DN cell model. Afterward, the miR-296 mimic and sh-SGLT2 were added to the DN cell model. Subsequently, the expression levels of SGLT2, collagen IV, TGF-*β*1, ACE, AT1, and VEGF in each group of cells were detected via qRT-PCR. circ_000166 could increase the mRNA expression levels of these factors in each group of cells. Overexpression of miR-296 and knockdown of SGLT2 could reverse the effects of hsa_circ_000166 on these factors (Figures 6(a)–6(f)). Cell apoptosis in each group was detected via flow cytometry. circ_000166 could increase apoptosis in each group. Overexpression of miR-296 and knockdown of SGLT2 could reverse the proapoptotic effect of hsa_circ_000166 (Figure 6(g)). In sum, both the overexpression of miR-296 and the silencing of SGLT2 reversed the progression of diabetic renal fibrosis regulated by circ_00016.

## 4. Discussion

Diabetic renal fibrosis is one of the most common causes of end-stage renal disease [19]. Many studies reported STZ-induced renal fibrosis in C57BL/6 mice [20, 21]. Our group also successfully constructed an animal model of diabetic renal fibrosis through STZ induction. Profibrotic activity is closely related to renal dysfunction [22]. Thus, promoting the renal fibrosis target of DN and preventing it are important.

Recent studies comprehensively explored the role of ncRNA in the process of gene expression. The circRNA-miRNA-mRNA network is closely related to the occurrence and development of numerous diseases, such as osteoporosis [23], cancer [24], and viral infections [25]. circRNA can act as a bridge between nc-RNA and c-mRNA. circRNA is produced by the reverse splicing mechanism during posttranscriptional processing and is expressed in large amounts in eukaryotic cells. circRNA can function by regulating RNA transcription and protein production, as well as by acting as a sponge for microRNA (miRNA).

Liu et al. [26] claimed that miRNA-296 promotes the healing of diabetic wounds by targeting SGLT2. Zhang et al. [27] reported that miRNA-296 can downregulate the expression of SGLT2 in lung cancer. Therefore, we suppose that miRNA-296 can regulate SGLT2 gene expression. SGLT2 plays a key role in the process of diabetic renal fibrosis. At present, SGLT2 inhibitors have become a new drug for the treatment of diabetes [28, 29]. SGLT2 inhibitors have been proven to have good metabolic characteristics [30] and can substantially reduce atherosclerosis events, hospitalization due to heart failure and cardiovascular diseases, total mortality, and the progress of chronic kidney diseases [31, 32]. However, the effects of circ_000166 on miRNA-296-SGLT2 remain unclear.

In this study, the circRNA-miRNA-mRNA network was used as an entry point to explore the role of circ_000166/miR-296/SGLT2 in the progression of diabetic renal fibrosis. Compared with those in normal controls, the expression levels of circ_000166 in the kidney tissues of mice with diabetic renal fibrosis significantly increased. Silencing circ_000166 could minimize kidney damage and decrease urine protein levels, thereby inhibiting the progression of renal fibrosis. Results of dual-luciferase report tests showed that circ_000166 and miR-296 were internally regulated. circ_000166 could act as the ceRNA of miR-296 and competitively bind to miR-296, resulting in increased expression of the SGLT2 gene regulated by miR-296. These results were verified via in vivo and in vitro experiments. The lentivirus and plasmid vector transfection technology was used to overexpress miR-296 and silence SGLT2. Results showed that DN renal fibrosis and apoptosis were significantly reduced. circ_000166/miR-296/SGLT2 may become a new target in the progression of diabetic renal fibrosis, which is important to delay the progression of this disease and prevent end-stage renal disease.

We focused on the circ_000166/miR-296/SGLT2 regulatory network as the research object. We found that the interaction of this network can reduce the expression of diabetic renal fibrosis-related factors. Hence, it may become an effective target for intervention in the treatment of diabetic renal fibrosis.

## Data Availability

The analyzed data sets generated during the study are available from the corresponding author on reasonable request.

## Supplementary Materials

Supplementary Materials Figure S1: histological image stained with hematoxylin and eosin (H&E) of kidney tissue. Results showed the structural derangements of tubular epithelia and interstitium in the DN model.
