# Supplementary figures and images for: circ_000166/miR-296 Aggravates the Process of Diabetic Renal Fibrosis by Regulating the SGLT2 Signaling Pathway in Renal Tubular Epithelial Cells
Source: Dis Markers. 2022 May 16;2022:6103086. doi: 10.1155/2022/6103086 (PMC9126678; doi:10.1155/2022/6103086)

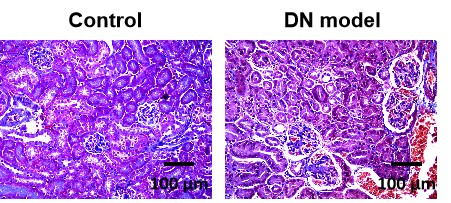

Supplement: Supplementary Materials — Figure S1: histological image stained with hematoxylin and eosin (H&E) of kidney tissue. Results showed the structural derangements of tubular epithelia and interstitium in the DN model. [file 6103086.f1.jpg]
